# A 32-channel parallel transmit system add-on for 7T MRI

**DOI:** 10.1371/journal.pone.0222452

**Published:** 2019-09-12

**Authors:** Stephan Orzada, Klaus Solbach, Marcel Gratz, Sascha Brunheim, Thomas M. Fiedler, Sören Johst, Andreas K. Bitz, Samaneh Shooshtary, Ashraf Abuelhaija, Maximilian N. Voelker, Stefan H. G. Rietsch, Oliver Kraff, Stefan Maderwald, Martina Flöser, Mark Oehmigen, Harald H. Quick, Mark E. Ladd

**Affiliations:** 1 Erwin L. Hahn Institute for MRI, University of Duisburg-Essen, Essen, Germany; 2 RF & Microwave Technology, University of Duisburg-Essen, Duisburg, Germany; 3 High-Field and Hybrid MR Imaging, University Hospital Essen, Essen, Germany; 4 Medical Physics in Radiology, German Cancer Research Center (DKFZ), Heidelberg, Germany; 5 Electromagnetic Theory and Applied Mathematics, Faculty of Electrical Engineering and Information Technology, FH Aachen – University of Applied Sciences, Aachen, Germany; 6 Faculty of Physics and Astronomy and Faculty of Medicine, University of Heidelberg, Heidelberg, Germany; New York University School of Medicine, UNITED STATES

## Abstract

**Purpose:**

A 32-channel parallel transmit (pTx) add-on for 7 Tesla whole-body imaging is presented. First results are shown for phantom and in-vivo imaging.

**Methods:**

The add-on system consists of a large number of hardware components, including modulators, amplifiers, SAR supervision, peripheral devices, a control computer, and an integrated 32-channel transmit/receive body array. *B*_*1*_^*+*^ maps in a phantom as well as *B*_*1*_^*+*^ maps and structural images in large volunteers are acquired to demonstrate the functionality of the system. EM simulations are used to ensure safe operation.

**Results:**

Good agreement between simulation and experiment is shown. Phantom and in-vivo acquisitions show a field of view of up to 50 cm in z-direction. Selective excitation with 100 kHz sampling rate is possible. The add-on system does not affect the quality of the original single-channel system.

**Conclusion:**

The presented 32-channel parallel transmit system shows promising performance for ultra-high field whole-body imaging.

## Introduction

Since the early days of MRI, a constant drive to higher main magnetic field strengths (B_0_) has existed. While the increase in B_0_ leads to a superlinear increase in SNR at higher magnetic fields [1, 2], the wavelengths of the electromagnetic fields at the corresponding Larmor frequencies decrease. At ultra-high field (≥ 7T), the wavelength in tissue is shorter than the diameter of a human torso. Consequently, substantial wave effects can give rise to inhomogenous spin excitations, which can lead to contrast artifacts and complete signal dropouts in the field of view [3–5]. Not only do these inhomogeneities affect the SNR, they also lead to varying contrast throughout the field of view, severely reducing the clinical utility of the resulting images.

To cope with the challenges introduced by the decreased wavelength at 7T and above, many different techniques have been proposed. Examples of these techniques are adiabatic pulses [6], RF shimming [7, 8], kT-points [9], 2D spokes [10], 3D tailored radiofrequency pulses [11], Transmit SENSE [12, 13], and TIAMO [14]. Although there are many differences between these techniques, most of them require a multi-channel transmit system. Moreover, the obtainable transmit power efficiency, specific absorption rate (SAR) efficiency, and pTx speed-up factor improve as the number of independent transmit channels increases [15].

Using 8- and 16-channel transmit systems, an increasing number of clinical feasibility studies at 7T have aimed at regions where imaging is hampered by severe transmit inhomogeneities, for example liver [16, 17], kidneys [18], prostate [19–21], female pelvis [22], the hip joints [23, 24], shoulder [25, 26], the heart [27], the spinal cord [28], and the breasts [29, 30], or they cover extended regions such as the complete lower extremities [31].

Most of these studies use local transmit arrays to enhance transmit efficiency, and many of these studies report a need for higher transmit power than what was available (generally 8 kW) despite the use of a local array. Such arrays that are placed either directly on or very close to the patient take up significant space in the patient bore of the magnet, reducing the maximum patient size. To get to a more clinical workflow, where a transmit body coil is placed further away from the patient, several stand-off arrays and arrays integrated into the bore have recently been proposed [32–34].

In this paper we present a 32-channel transmit system add-on for 7T, including modulator system, power amplifiers, and SAR supervision system. The 32-channel body array is integrated into the MRI system by placing it in the gap between the gradient coil and liner of the patient tunnel, similar to body coils at lower field strengths and thereby enabling a workflow very close to conventional clinical MRI.

## Methods

### Ethics approval

For in-vivo measurements, informed consent was obtained from both participants included in the study. In vivo studies were approved by the institutional review board of the University Clinic Essen. Approval nr. 16 7214BO.

### Original MR system

The vendor-provided MR System in this study is a Magnetom 7T with an AS095 gradient coil (Gradient strength 38 mT/m, slew rate 200 mT/m/ms; Siemens Healthineers, Erlangen, Germany). The nominal field of view is 50 cm in all directions, and the system has 32 receivers in total. It is equipped with the “Step 2” 8-channel transmit system, which was not used in this work.

### Overview of system add-on

**Fig 1:**
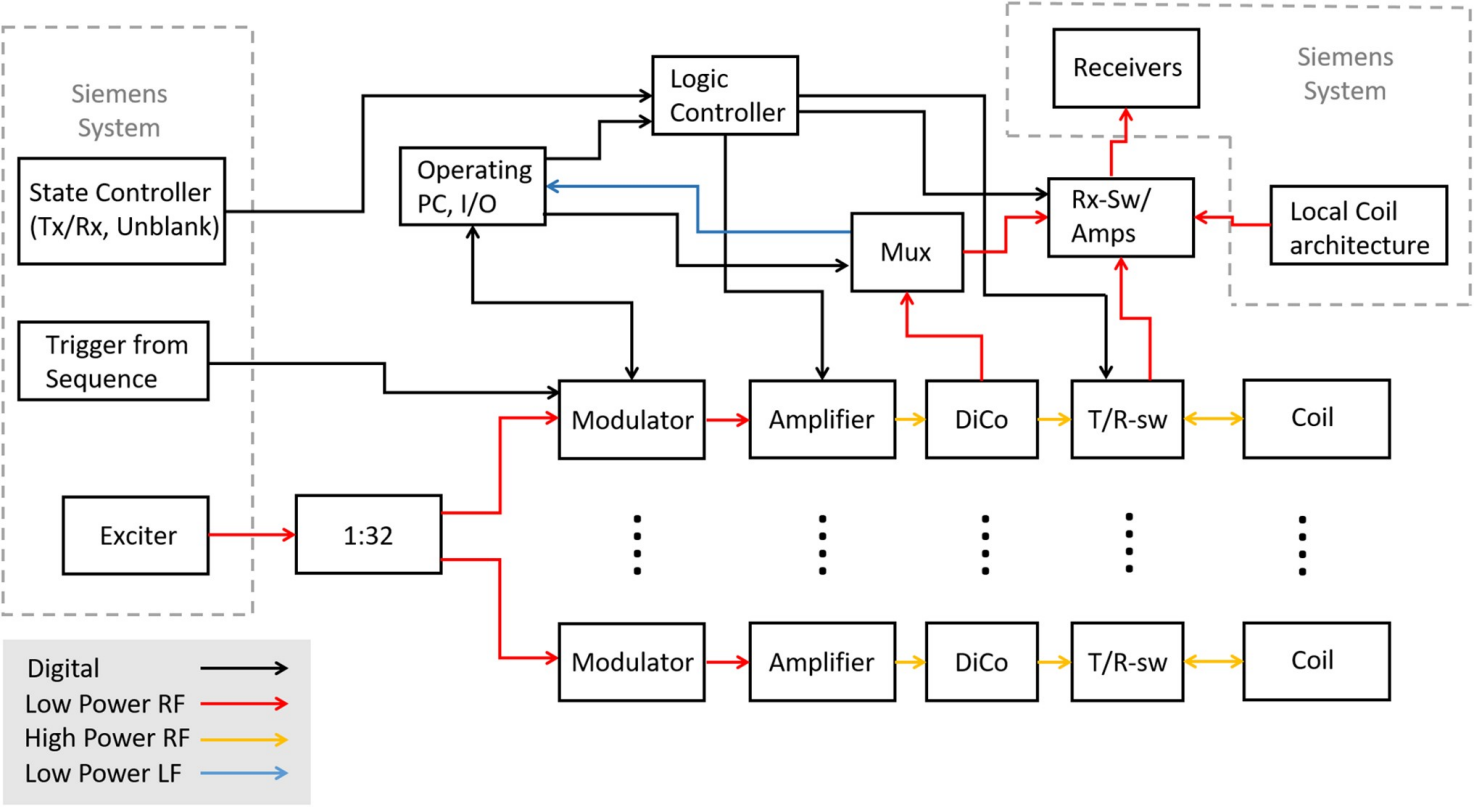
Simplified schematic overview of the add-on system. Arrows show the direction of communication. Black arrows indicate digital signals, red arrows indicate low-power RF signals, orange arrows indicate high-power RF signals, and blue arrows indicate low-power low-frequency analog signals. The dashed gray lines indicate that the enclosed components are part of the original system.

Fig 1 shows a schematic overview of the add-on system. The system only uses 4 input signals for synchronization with the original system, three digital signals and one analog signal. The digital signals are 1 bit for transmit or receive state, 1 bit for the unblank signal for the transmit amplifiers, and 1 bit as a trigger from the sequence to set the modulators to the next pre-programmed state. The analog signal is the RF signal coming from the exciter, which is the signal that would be sent to the power amplifier in the single-channel mode of the original system.

The signal from the exciter is split up and fed to the modulators. In the original single-channel system, this signal is directly fed into the vendor power amplifiers that are combined into a single high-power output. The output of the modulators is connected to the power amplifiers in the magnet room. The high-power signals from the amplifiers are fed to the RF coil via directional couplers (DiCos) and transmit/receive (T/R) switches.

To be able to switch between reception with local coils or the body array, the receive chain of the system is modified to include RF switches. This is the only change to the vendors transmit or receive path.

The RF signals for forward and reflected power are taken from the DiCos and fed into a multiplexing power supervision unit (MUX). This unit feeds a portion of the forward power into the receive chain and sends a logarithmic baseband voltage signal to the operating PC system for amplitude supervision.

The operating PC is used to pre-program the modulators with a set of states before a sequence is run. Using the trigger from the sequence, the modulators are successively switched to the next state.

The logic controller receives the information about whether the MR system is in transmit or receive mode as well as the unblank signal and distributes the signals across the add-on system.

### Modulators

The custom-built modulator units [35] use AD8345 (Analog Devices, Norwood, MA, USA) IQ modulators to modify the exciter signal. The exciter signal is fed into the local oscillator input at a very low power (<-30 dBm), to ensure that changes in the input are transferred linearly to the output, since in normal operation the input power for the AD8345 is far beyond the 1 dB compression point. The analog input voltages for I and Q are provided by MAX5100 8-bit digital-to-analog converters (DACs) (Maxim Integrated Products, Sunnyvale, CA, USA). These DACs have an input register that allows programming them to a new value before switching them to that new value by a trigger signal. Due to the rise time of the output operational amplifiers of the MAX5100, output settling time is up to 3 μs, depending on the degree of change of the outputs. The modulator system is divided into two half systems with 16 modulators each. Each half system is connected to a 16-bit parallel bus provided by a digital I/O card. On a trigger from the pulse sequence of the MRI system, a new bit pattern is loaded into the input register of the DACs. After all input registers are filled with the new pattern, a trigger is issued to simultaneously move the data from the input register to the DACs, changing the modulator state. To account for delays, the first trigger from the sequence is sent 10 μs before the start of the RF pulse. Adjustment of the data rate of the bus allows to fine tune the delay between the trigger from the MRI pulse sequence and the point in time when the DACs change their output.

**Fig 2:**
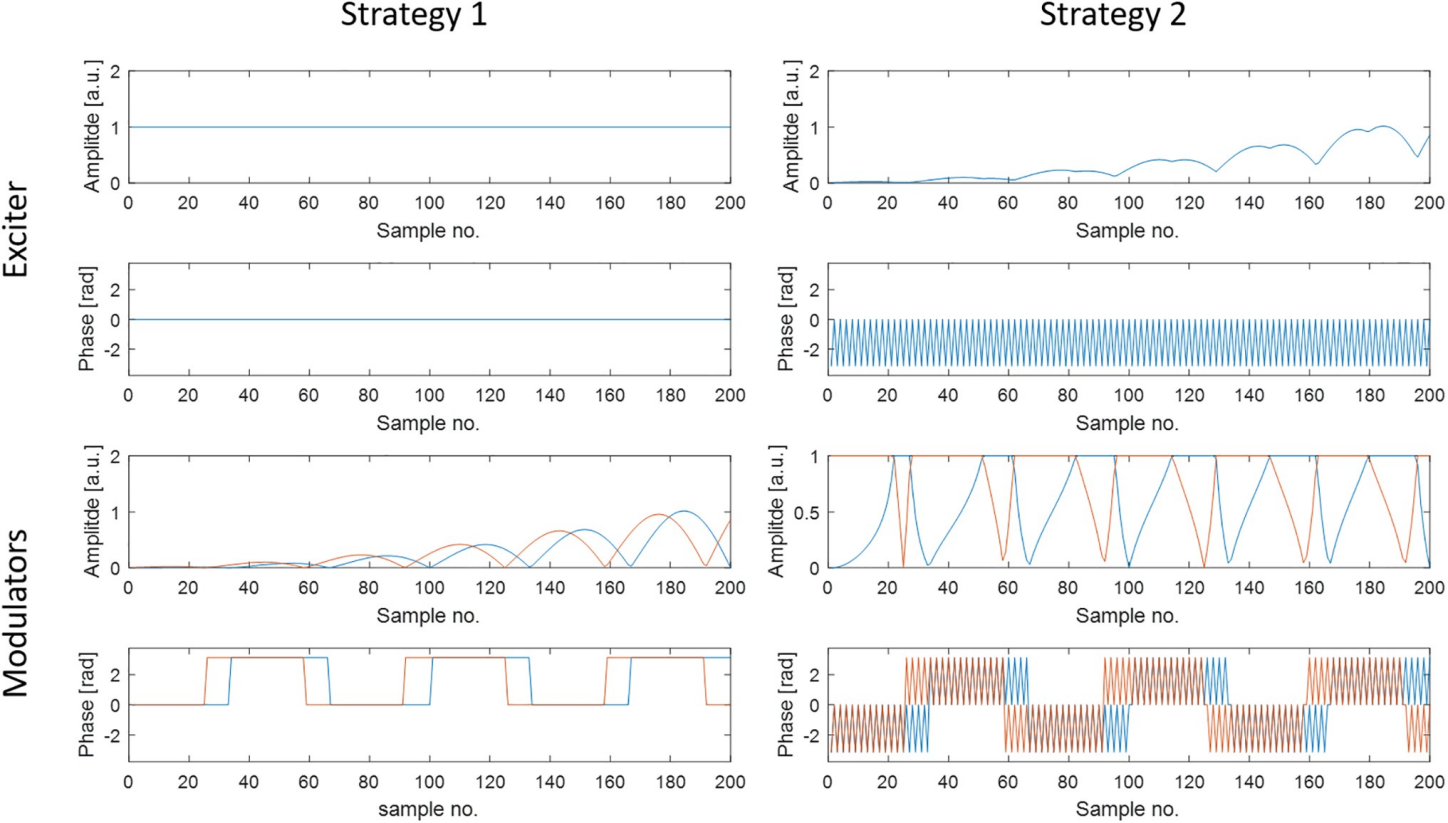
Simplified representation of two modulation strategies using two channels. Strategy 1 plays out a rectangular pulse on the exciter and performs the entire modulation with the IQ modulators. Strategy 2, which is used in the implemented system, uses a pre-modulated signal from the exciter to always use the full dynamic range of the modulators, as well as a 180° change in phase from sample to sample to minimize the effect of systematic errors in the modulators.

**Fig 3:**
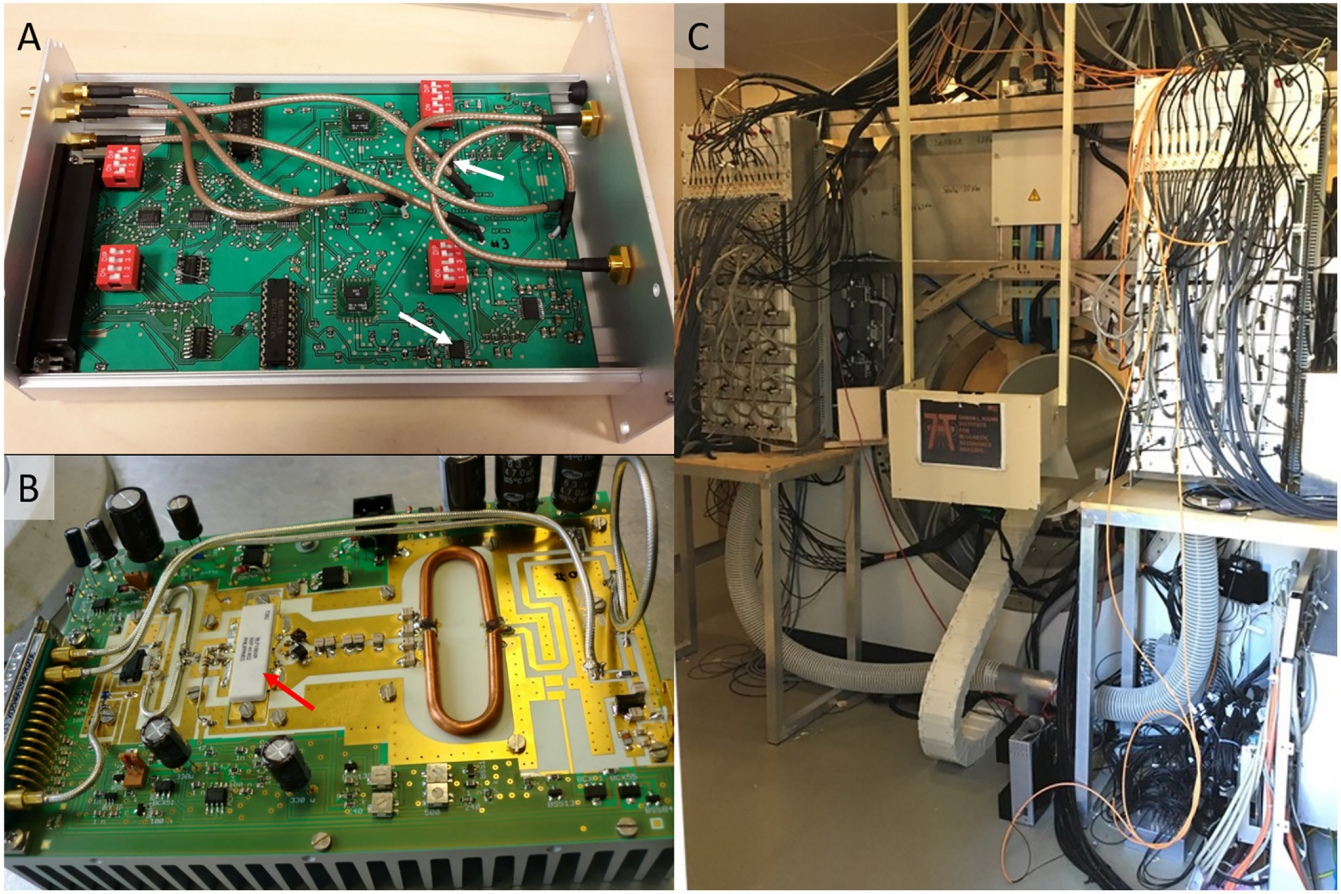
Pictures of modulators and amplifiers. A) Modulator unit containing two modulators. The position of the modulator ICs is indicated by the white arrows. B) High-power amplifier. The red arrow points to the final amplifier stage LDMOS IC. C) Power amplifier racks at the back of the magnet; each rack accommodates 16 amplifiers.

To account for small offsets in the I and/or Q channel and other systematic errors after calibration, the modulation strategy for pTx is performed as follows. Instead of playing out a rectangular pulse on the exciter and performing the modulation completely with the modulators (“Strategy 1” in Fig 2), the exciter plays out a signal in which each sample is the maximum amplitude across all channels. In this way, a variable dynamic range is achieved for the 8-bit modulators. Errors due to offset in the I and/or Q channel are further decreased by shifting the exciter phase by 180° for each sample, which is accounted for in the phases the modulators apply (“Strategy 2” in Fig 2). Changing the phase each sample by 180° leads to alternatingly adding and subtracting the offset. Fig 3A shows a picture of a modulator unit containing two modulator circuits.

The modulators are run at a 100 kHz sampling rate, in accordance with the sampling rate of the system’s gradients. Signals with higher baseband frequency can be generated by the system’s exciter, and these are fed through the modulators, providing extra pulse form control in addition to the modulators.

### High-power amplifiers

The custom-built high-power amplifiers are designed to be used close to the magnet in order to minimize cable losses [36]. Each amplifier consists of three amplifier stages with an overall gain of slightly more than 60 dB. The first stage is a MGA-31189-BLKG (Broadcom Inc., San José, USA), the second stage is a MRF6V2010NR1 (NXP Semiconductors N.V., Eindhoven, The Netherlands), and the final amplification stage of each amplifier is a BLF188XR power LDMOS transistor (Ampleon BV, Nijmegen, The Netherlands). With the final stage driven in AB mode, the amplifier has a peak output of 1 kW. Without an unblank signal, the bias voltages of the last two amplifier stages are set to 0 V, and the 50V power supply for the final stage is cut off via a high power MOSFET. This ensures that no power can be transmitted without an unblank signal present. Each amplifier contains a 61 mF capacitor array and a high-power voltage regulator to supply peak power during transmission. DC power supplies for the whole system can continuously deliver 6 kW in total. In case of an emergency shutdown, the capacitor arrays are discharged through load resistors inside the power supplies.

Fig 3B shows a picture of an opened power amplifier. Fig 3C shows the position of the two power amplifier racks at the back of the magnet; the second rack is just visible at the right border.

To ensure stable behavior independent of reflected power, circulators (JCC0296T0298N20-GIG, JQL Electronics Inc., Rolling Meadows, USA) are connected to the outputs of the amplifiers. These circulators have an insertion loss of 0.15 dB. Since circulators are highly magnetic, the circulators are placed just outside the magnet’s passive shielding, resulting in an extra cable length of 7 m in total (Aircell 7, SSB-Elektronik GmbH, Lippstadt, Germany), which is shorter than the minimum cable length necessary to place the amplifiers outside the magnet room at our site (14 m). The overall attenuation of these cables is 0.78 dB. The air to cool the amplifiers is taken from the room’s air-conditioning input duct by two radial fans in series and delivered via a tubular system over a distance of 15 m.

Due to the strong currents on the power supply cables of each amplifier rack, the ground of the DC power supplies is only connected to a common ground directly at the amplifier housing, ensuring that the current of up to 60 A on the feed line is identical in both directions and resulting in no net force on the cable as it crosses the magnetic field.

### Directional couplers

The custom-built directional couplers are fabricated in strip-line technology with 1.5-mm-thick FR4 substrate material separating the strips from the top and bottom copper layers. The distance between the conductors is 3.4 mm (center to center) and the width of 0.94 mm was calculated numerically using CST Microwave Studio (CST AG, Darmstadt, Germany). The coupled length is 69 mm, resulting in a coupling of -29 dB. The output is further reduced by 20 dB attenuators for both the forward and reflected output signals. To maximize directivity, the coupled lines are terminated by a variable resistor and a trimmer capacitor, which were fine-tuned after production. The insertion loss is 0.2 dB. The couplers are placed in two boxes with 16 DiCos each on the tables next to the amplifier racks.

### Power supervision

The power supervision units get the forward and reflected signals from the directional couplers. A power splitter in the forward path feeds half of the power directly to the receive chain while the other half as well as the reflected signal are each fed into a logarithmic amplifier AD8307 (Analog Devices, Norwood, MA, USA). With the use of the logarithmic amplifiers, the forward and reflected signals are translated into voltage signals proportional to the logarithm of the input power. Since the envelope of the RF power only changes slowly, the result is a low-frequency signal that is multiplexed and sent to two NI5751 digitizer cards (National Instruments, Austin, TX, USA) with 16 analog inputs each that are connected to the operating PC I/O system. Here, the forward power in each channel is sampled every microsecond for amplitude supervision [37]. The software is implemented in a LabView environment (National Instruments, Austin, TX, USA). If the maximum allowed input power is exceeded, the supervision prevents the unblank signal from being conveyed to the amplifiers.

### Receive chain

**Fig 4:**
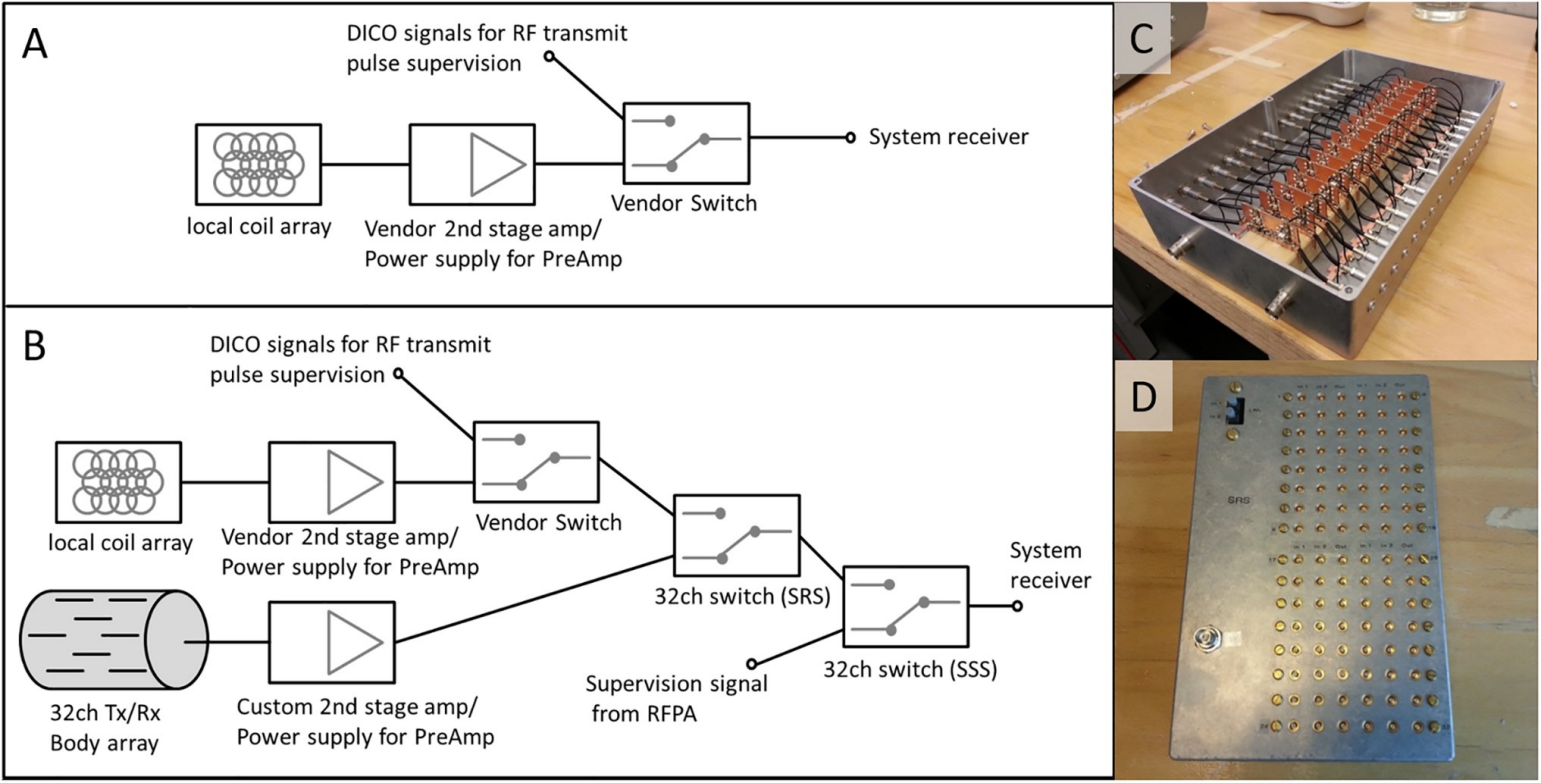
Overview of the receive chain. A) shows the original receive chain as provided by the vendor. B) shows the receive chain after including custom components. C) shows the custom second-stage amplifier with bias tee for the on-coil pre-amps. D) shows one of the 32-channel switches (SRS and SSS).

To enable automated switching between local and body coil reception as well as to feed power supervision signals into the system’s receivers, several changes were made to the receive chain [38]. Fig 4A shows the original receive chain of the system as provided by the vendor. It contains a second-stage receive amplifier as well as a switch to allow input of power supervision signals from the vendor-provided 8-channel transmit system. Fig 4B shows the changes made to the receive chain. A custom-built second-stage receive amplifier and power supply for the on-coil pre-amps is used for reception with the body coil. The second-stage amplifier (Fig 4C) uses an MGA-62563-BLKG (Broadcom Inc., San José, USA), has a gain of 22 dB and a noise figure of 1 dB. The input 1 dB compression point is -3.5 dBm. The power supply for the on-coil pre-amps and the second-stage receive amplifier can be switched off during transmit mode. This helps to protect the pre-amplifiers and reduces spurious signal in the receive path that might affect the power supervision, reducing the demand on the off state-isolation of the following switches. A custom-built 32-channel 2:1 switch (SRS, Fig 4D) is used to select between local and body coil reception and another switch (SSS) is used to integrate the signals from the directional couplers for transmit supervision. These switches are identical. Each channel contains three MASWSS0155 GaAs SPDT switches (M/A-COM Technology Solutions Inc., Lowell, MA, USA) to achieve an off-state isolation of better than -55 dB and an insertion loss of better than -0.6 dB.

### Logic controller

The logic controller receives the unblank and Tx/Rx signals from the original system. Furthermore, it accepts signals from a digital I/O card from the operating PC. These signals go through a logic circuit and are then distributed to the power amplifiers (unblank signal), to the receive chain (Rx amplifiers on/off, SRS and SSS switching states), and to the T/R switch controller circuitry (transmit/receive state, tune/detune). All logic is implemented in hardware using standard logic gate ICs to reduce the probability of failure as compared to microcontrollers or embedded systems.

### Body coil

**Fig 5:**
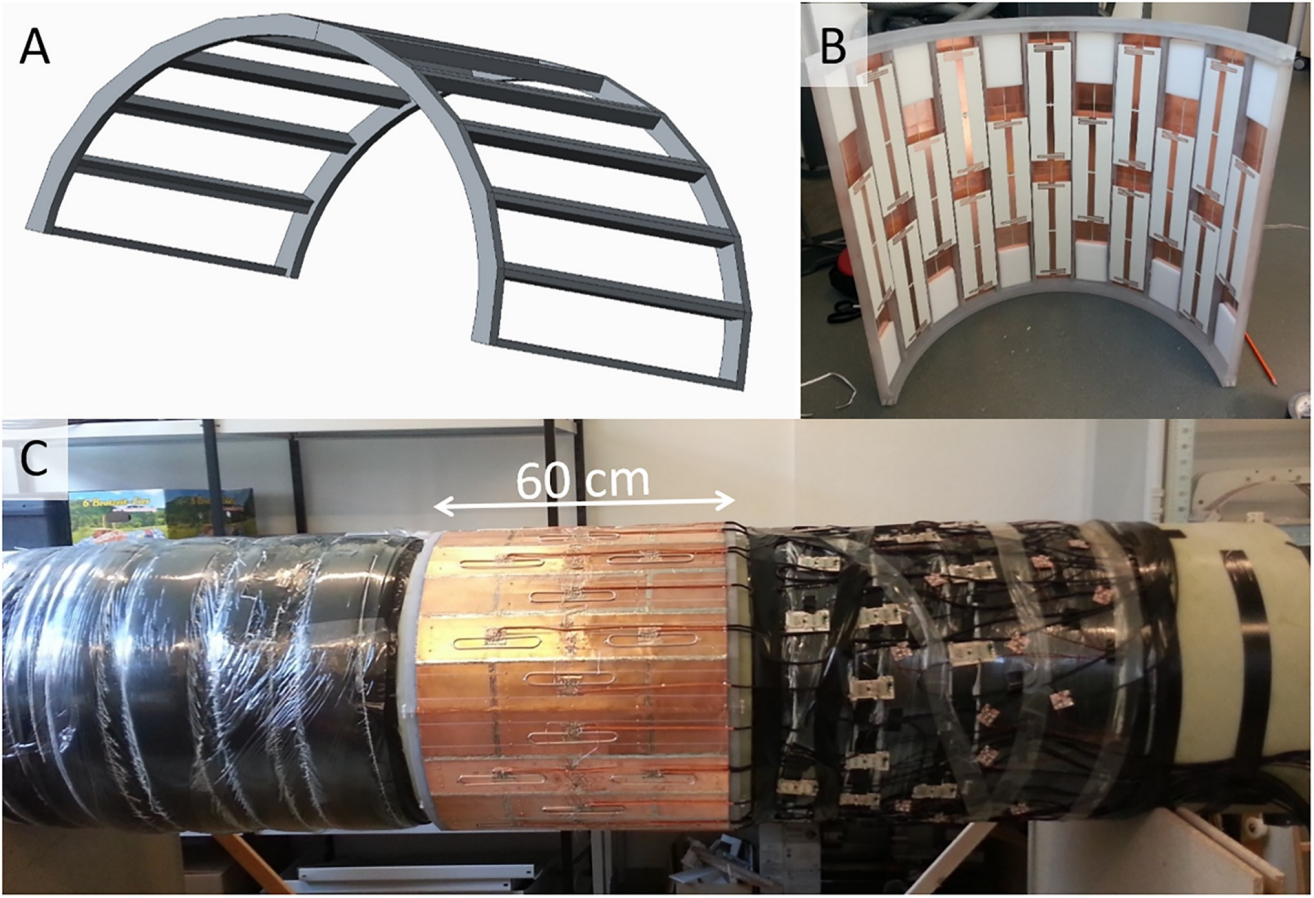
32-channel body coil. A) shows the polycarbonate frame that defines the mechanical structure. B) shows an inside view of one half of the array. 16 micro strip-line elements with meanders are visible. C) shows the two halves of the array mounted on the bore liner, with the T/R switches on the bore liner to the right side of the image (service end).

Since the 7T MR system is equipped with the AS095 gradient coil, there is a space of 34 mm between the bore liner (615 mm diameter) and the gradient coil (683 mm diameter). This space is used to accommodate the integrated body array [39]. The body array is mounted on a polycarbonate frame (Fig 5A) that consists of two halves forming a cylinder with 22 outer faces. The length of the cylinder is 60 cm and the inside of the polycarbonate frame rests directly on the bore liner. The longitudinal bars stand off from the bore liner so that the coil elements have a distance of about 8 mm from the bore liner.

The coil elements are micro strip-line elements with meanders, which were chosen because of their good intrinsic decoupling when lying side by side even in low load conditions [40]. The elements have a length of 25 cm and are arranged in 3 rings: two outer rings of 10 elements and one inner ring of 12 elements (Fig 5B). The distance between the ground plane and the strips is 20 mm as was used in previous studies [32, 40]. The ground plane completely encloses the coil. It is made of 0.127-mm-thick RO3010, both sides clad with copper. The copper of both sides is slotted in the longitudinal direction in such a way that the copper strips of the inner and outer layers overlap. In this way, RF currents can flow in the circumferential direction, while eddy currents from the gradient fields are effectively blocked. For mechanical stability the ground plane is glued with epoxy to a 1-mm FR4 sheet on the inner surface.

**Fig 6:**
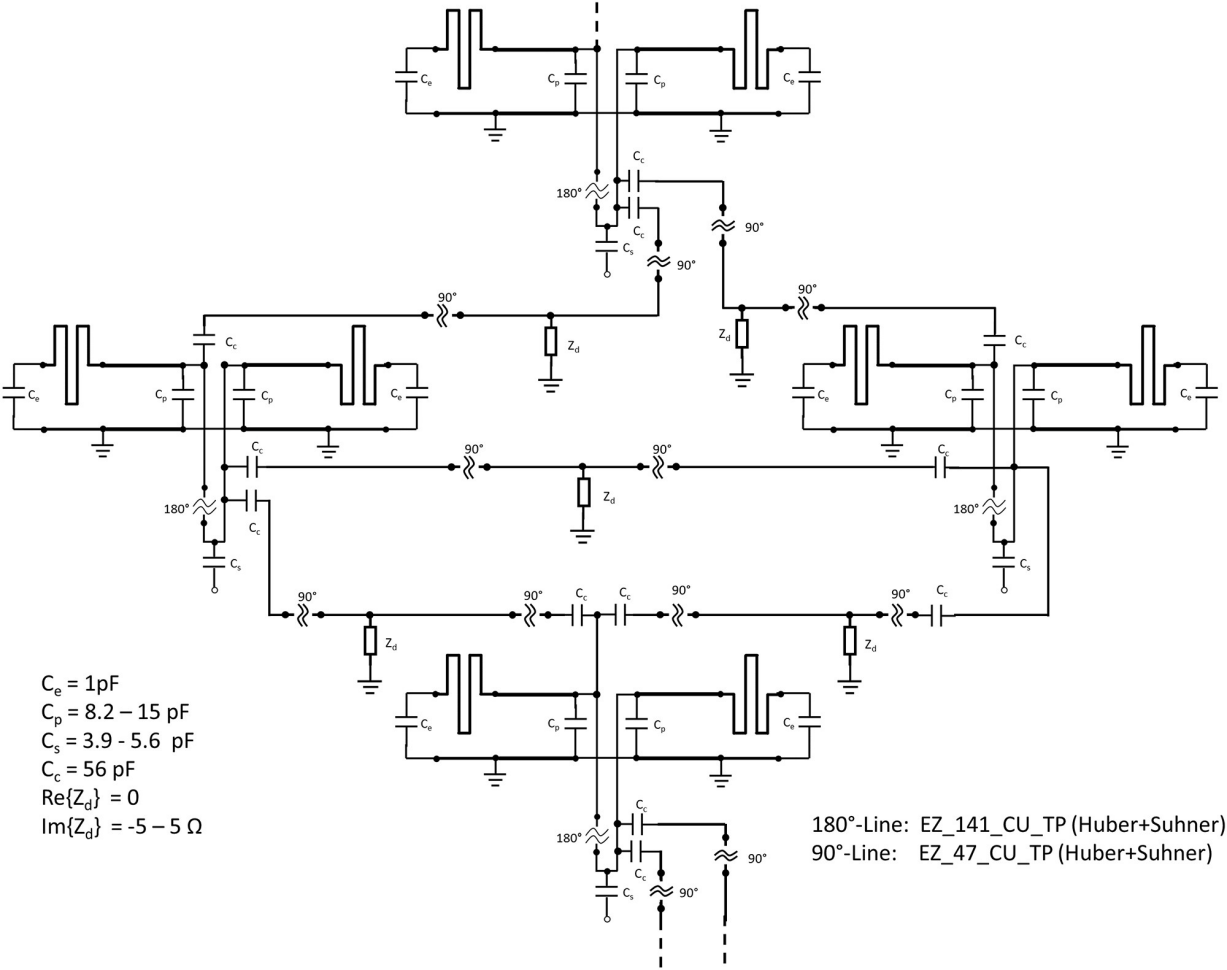
Decoupling network. Schematic of the body array showing four elements including the decoupling network for diagonally placed and head-on elements. C_e_ is used to set the correct current distribution on the element, C_p_ and C_s_ are the parallel and series capacitors of the matching network, and C_c_ and Z_d_ are part of the decoupling network. The 180°-lines are used for balancing the feed; the 90° delay lines are used as part of the decoupling network. These lines are semi-rigid cables from Huber+Suhner AG, Herisau, Switzerland.

While micro strip-line elements with meanders are well decoupled when lying next to each other, additional decoupling is needed for diagonally adjacent elements and elements placed head-on. Fig 6 shows an excerpt of the schematic of the body array. Decoupling is done using 90° delay lines connected through a very low impedance to ground. These lines provide another path for coupling that reduces overall coupling due to a different phase compared to the parasitic coupling via air and tissue. The two halves of the array are not electrically connected other than the connection of the ground plane.

The elements are connected to custom-built, compact T/R switches with a design similar to [41]; the only difference to the published design is that all components were placed on one side of a 81-mm by 42-mm PCB rather than using a folded layout to achieve a flatter profile. An appropriate length of low-loss cable (Aircell 5, SSB-Electronic GmbH, Lippstadt, Germany) transforms the impedance of the pre-amp to ensure pre-amp decoupling. The T/R switches introduce an attenuation of 1.1 dB. The Aircell 5 cables on the bore liner, connecting the T/R switches with the coil elements and the directional couplers, have an overall attenuation of 0.58 dB.

Detuning of the elements is performed with dedicated detuning boards containing PIN diodes that produce an RF short circuit of the transmit cable to ground when a forward current is applied. These detuning boards are placed in the transmit chain between the power amplifiers and the T/R switches with an appropriate length of cable to transform the short circuit to an impedance that detunes the elements.

### EM simulations

A detailed simulation model of the prototype coil was constructed for validation purposes and safety assessments. The model includes coil elements, the supporting frame, and the MR environment, i.e. RF shield, cryostat, bore liner, and patient table with a length of 200 cm to account for wave propagation [42].

Time-domain simulations were performed in CST Studio Suite 2016 (CST AG, Darmstadt, Germany) using the finite integration technique (FIT). The simulation domain was discretized with a hexahedral grid and 105 million mesh cells. A finer resolution was used inside the coil volume to account for coil details and a coarser mesh was used outside the coil volume. Tuning and decoupling of the coil elements were performed in a circuit co-simulation. All cables between the coil elements and the directional couplers were considered.

For coil validation, a homogeneous body-size phantom filled with tissue simulating liquid (*ɛ*_*r*_*’* = 45, *σ* = 0.55 S m^-1^) was simulated in the center of the coil. The phantom is a roughly oval-shaped cylinder with a length of 50 cm, a width of 34 cm, and a height of 20.7 cm; the filling volume is 32 liters. *B*_*1*_^*+*^ maps for excitation of individual channels and CP+ and CP2+ mode were exported for measurement validation. The simulated *B*_*1*_^*+*^ maps were compared to maps acquired with the B1TIAMO [43] technique.

For safety assessments, a male and female anatomical body model with a resolution of 2x2x2 mm³ [44] were placed in head-first supine position on the table in the coil center. The normalized electric field distributions were exported and 10g-averaged Q-matrices were calculated for every voxel in the body model. Subsequently, the Q-matrices of both body models were compressed together using the ‘virtual observation point’ (VOP) algorithm with a maximum overestimation of 10% to 420 VOPs [45]. No differentiation was made between the extremities and the rest of the body, so the head and trunk local SAR limit, which is more restrictive, was used for the extremities as well.

### Quality assurance

A routinely applied quality assurance (QA) protocol [46] was used to investigate the influence of the add-on system on the performance of the original system. A 1-channel Tx / 32-channel Rx head coil (Nova Medical, Inc., Wilmington, USA) was loaded with a tissue simulating phantom (*ɛ*_*r*_*’* = 55, *σ* = 0.6 S/m). The QA protocol includes tests to check for the performance of the local RF coil as well as gradient and system stability. The proper function of the RF head coil was verified by *B*_*1*_, SNR, and coupling measurements (i.e. noise correlation). Unwanted noise sources and RF spikes were searched for with noise measurements. System stability was measured with high-duty-cycle EPI imaging (TR 1000 ms, TE 30 ms, echo spacing 0.54 ms, BW 2112 Hz/pixel, 64 phase-encoding steps, 16 slices, FOV 220 mm, 3.4x3.4x2 mm^3^, 3x 250 measurements), and stability parameters such as SNR, Signal-to-Fluctuation-Noise Ratio (SFNR), signal drift, fluctuation, and ghosting were calculated according to the recommendations of the FBRIN consortium [47].

### Imaging experiments

In all imaging experiments the 32-channel body array was used for transmission as well as reception.

For phantom measurements a torso phantom filled with tissue simulating polyvinylpyrrolidon solution (*ɛ*_*r*_*’* = 45, *σ* = 0.55 S m^-1^) was used that has the same dimensions as the one used in the simulations.

For in-vivo measurements, informed consent was obtained from both participants included in the study. All imaging experiments with the volunteers were performed with an input power not exceeding the maximum allowed input power for the worst-case shim set used within an experiment, with an extra safety margin of a factor of 2. This was done to ensure that even if an erroneous trigger signal would alter the shim setting, the SAR would remain within the allowed limits.

*B*_*1*_ mapping was performed using the B1TIAMO method [43]. Absolute *B*_*1*_ maps were acquired in a central transversal slice in the torso phantom and compared with simulation data. Furthermore, *B*_*1*_ mapping was performed in a human volunteer (1.86 m, 80 kg) in an axial slice through the kidneys and a coronal slice through the center of the torso with the CP+ and CP2+ modes. In this case we define the phase for each element via its respective geometrical angle in the xy-plane.

Structural imaging was performed in a healthy volunteer of above-average size (1.85 m, 95 kg). A 2D gradient echo sequence with 1.1-mm in-plane resolution and 5-mm slice thickness, a TR of 50 ms, and a TE of 6.1 ms was used to acquire slices with a 50-cm field of view. Data sets from the head to the thighs were acquired in three stations with approximately 10 cm overlap. TIAMO [14] with the CP+ and CP2+ modes was used for transmit homogenization. For the slices in the abdominal region, the volunteer was asked to place the arms above the head. Each image was acquired during a breath hold. The vendor’s gradient distortion correction was used.

To demonstrate the selective excitation capability of the system, four different patterns were excited in the torso phantom: the letters “ELH”, the logo of the Erwin L. Hahn Institute for MRI, a checker board, and the logo of the German Cancer Research Center (“DKFZ”). The pulse length was 5.36 ms (536 samples) with a variable density spiral for a 64 x 64 voxel target in 2D spatially selective excitation, implemented in a 3D gradient echo sequence with 128 x 128 in-plane voxels. The sampling rate for the transmit pulse was 100 kHz.

## Results

Implementation of the add-on system did not have detectable influence on the QA parameters of the system apart from a 1.2 dB reduction in received signal due to the switches integrated in the receive chain. Since these switches are behind the second receive amplifier stage, no change in SNR was detectable. Neither an immediate impact after integration nor a longer-term drift during the following 6 months was found; system users did not report any impairments in image quality that could be correlated with the integration of the add-on system.

The amplifiers were successfully tested to play out 10 ms pulses at full peak power. Duty cycle was limited to 10% or smaller to prevent overheating of the amplifiers.

**Fig 7:**
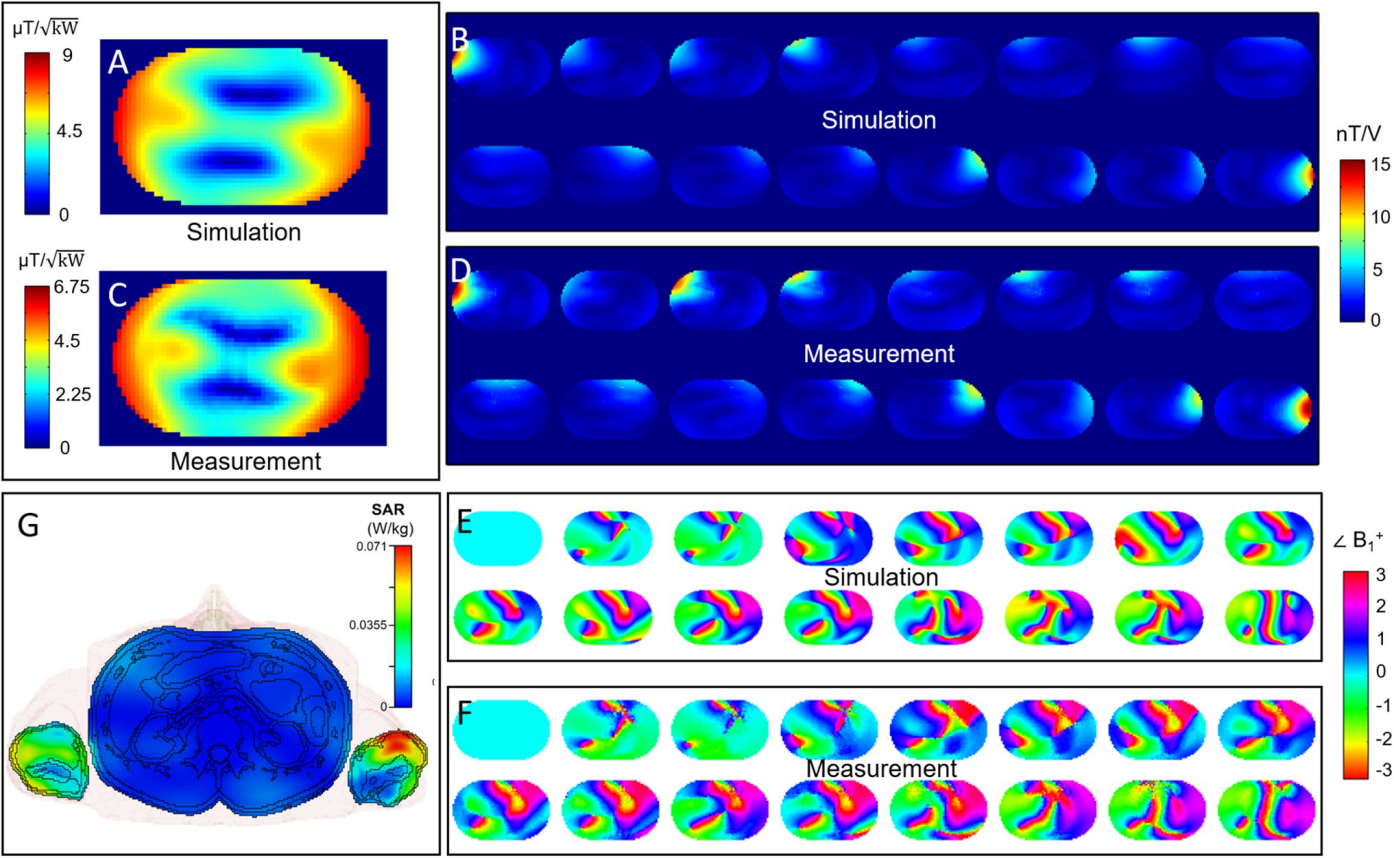
Phantom B_1_ maps and SAR distribution. Comparison of simulated *B*_*1*_^*+*^ maps (A,B,E) and measured *B*_*1*_^*+*^ maps (C,D,F) in the body-sized phantom. Please note that only half of the channels are shown for better depiction. G shows an axial cut through the body model at the position of the highest SAR in the CP+ mode, with the SAR distribution normalized to 1 W total input power as an overlay.

Fig 7 shows a comparison of the simulated and measured *B*_*1*_^*+*^ maps in the torso phantom for the CP+ mode (A, C) and the first 16 individual elements of the body array (B, D, E, F). The phase maps are normalized to the phase of the first channel. The maps show good agreement qualitatively and quantitatively in the absolute values (A, B, C, D) as well as in the depicted phase (E, F). The single-channel maps show good agreement even in areas far away from the transmitting elements. The normalized root-mean-square error for the CP+ mode is 16.6%. Further values are shown in [43]. With the shim shown in Fig 7C, a *B*_*1*_^*+*^ amplitude of 13 μT can be reached in the center of the phantom at full peak power. Fig 7E shows an axial cut through the body model at the position of the highest SAR in the CP+ mode, with the SAR distribution as an overlay. The highest SAR in the case of the CP+ mode occurs in the left arm. The values are normalized to 1W total accepted power.

**Fig 8:**
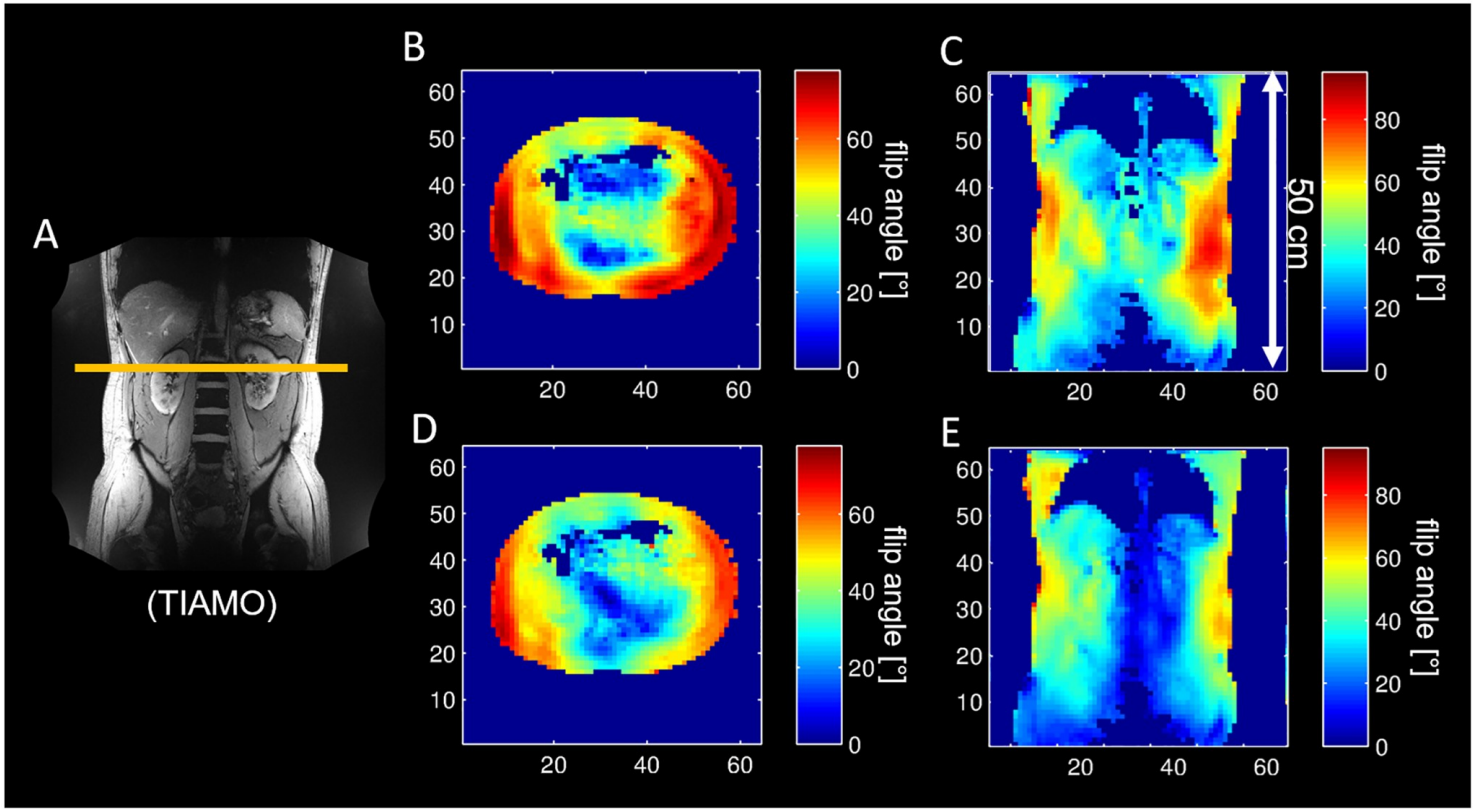
In-vivo flip angle maps. In-vivo flip angle maps with TIAMO image (A) for orientation. The maps show the CP+ (B, C) and CP2+ (D, E) modes in transverse (B, D) and coronal (C, E) orientation. The maps (C,E) have a field of view of 50 cm in z-direction.

Fig 8 shows in-vivo flip angle distributions for the CP+ and CP2+ modes of the body array. Fig 8A shows a coronal structural image including the position of the transverse slice for orientation. Fig 8B and 8C show the CP+ mode in transverse and coronal orientation; Fig 8D and 8E show the CP2+ mode in the respective orientations. The flip angle is fairly constant in z-direction over a 50 cm field of view.

**Fig 9:**
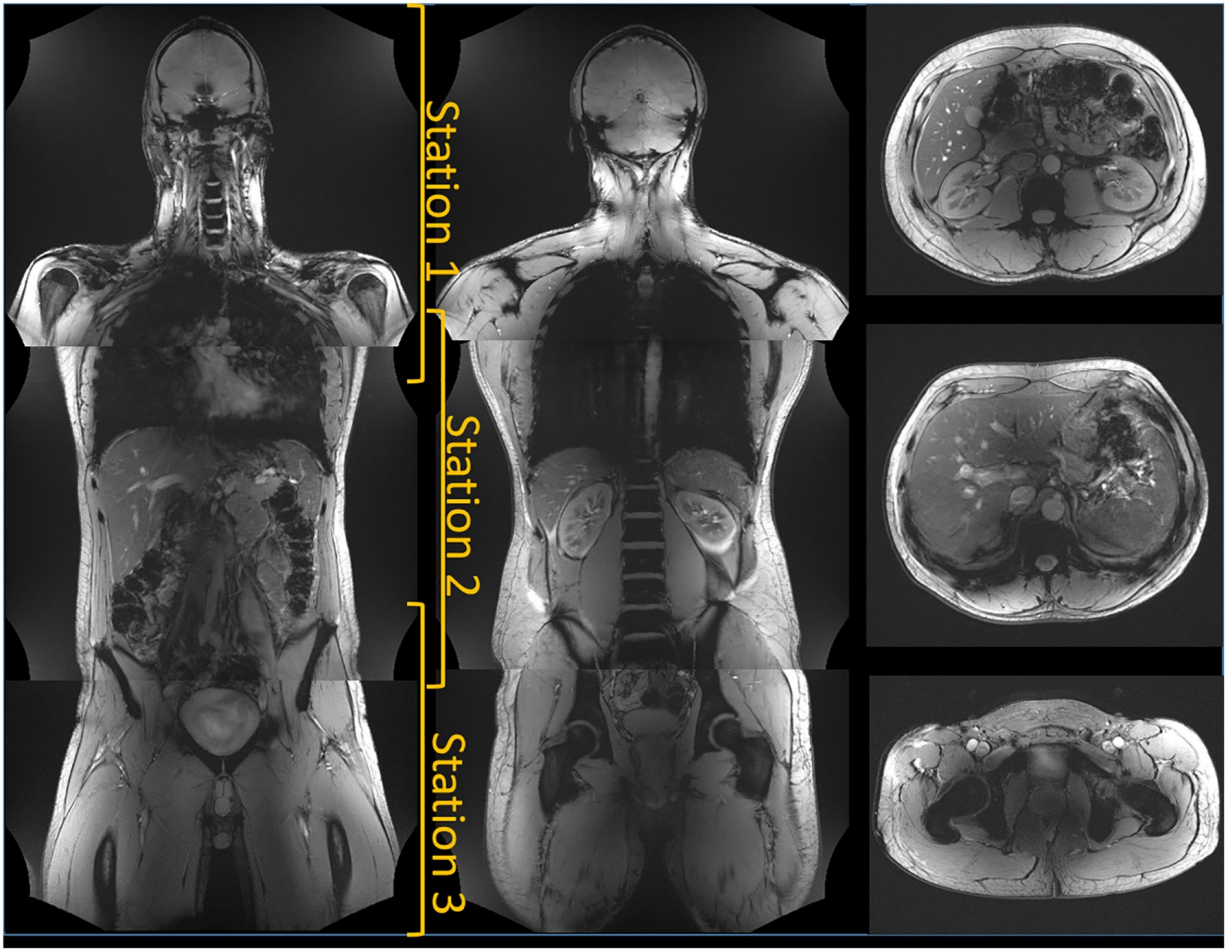
In-vivo images of a large volunteer using TIAMO. Three overlapping stations of 50 cm were acquired. The left images show coronal views, the three images on the right show transverse views of the same volunteer. All images were acquired with the body coil in Tx/Rx mode.

Structural body images are presented in Fig 9. Due to the capability of both the gradient and RF coil to cover a 50-cm field of view, the head, torso, and thighs of a large volunteer could be imaged in three stations. The images show good uniformity in contrast, and no complete signal dropouts are visible.

Both volunteers had undergone many 7T examinations in the past and reported that the examination was very comfortable compared to examinations with local transmit coils.

**Fig 10:**
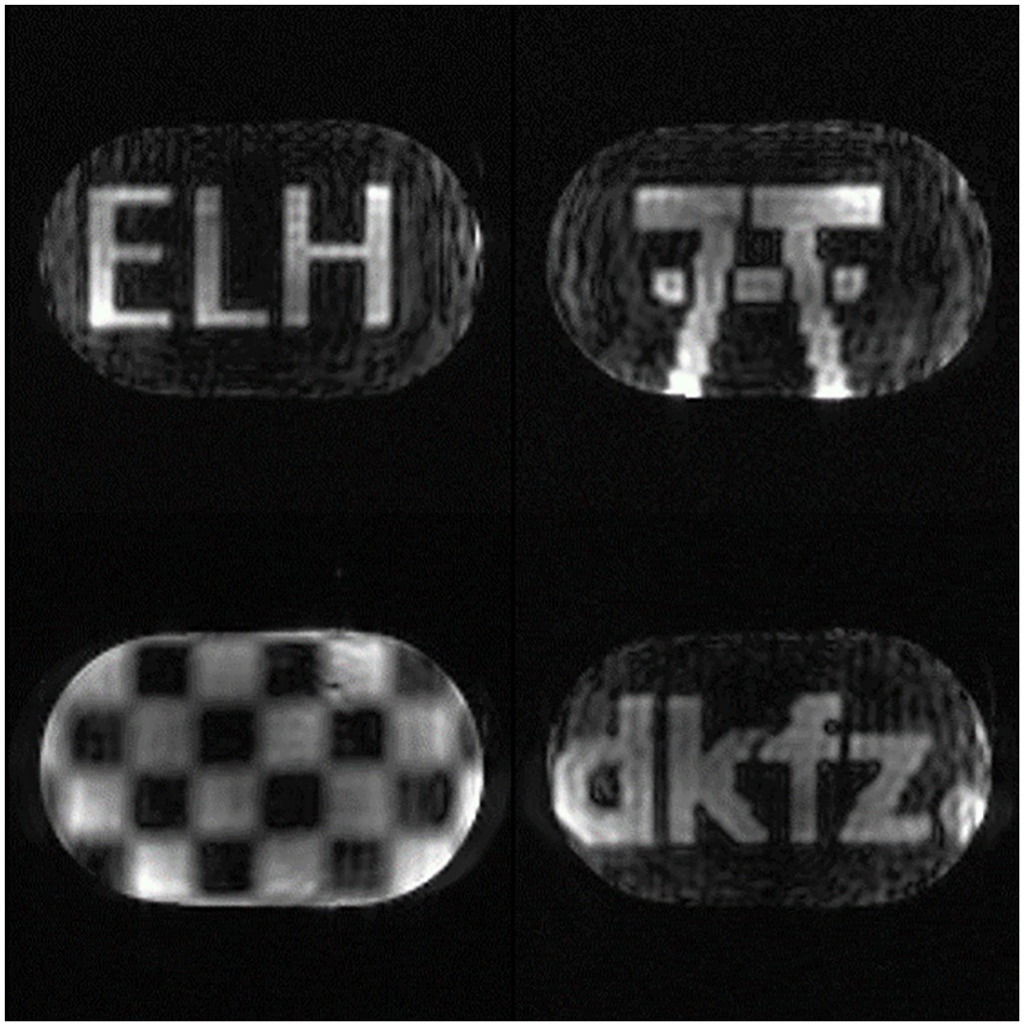
Selective excitation. 2D spatially selective excitation in a body-sized phantom using the 32-channel Tx/Rx body coil. The transmit sampling rate was 100 kHz.

2D selective excitation examples are shown in Fig 10. The images show the central slice of the 3D data set for which the pulse calculation was performed. The patterns show sharp edges and only low residual background excitation.

## Discussion

The results presented here show the feasibility of the presented system. Implementation is straightforward since only very few interfaces are necessary. Only one analog and three digital signals have to be taken from the original MRI system to make the add-on fully functional. In comparison to the development of complete consoles [48, 49], which might pose more powerful tools, the big advantage is the use of the standard vendor software that clinicians and researchers are accustomed to. For simple RF shimming with the add-on system, the vendor-provided sequences can remain completely unchanged. When selective excitation is used, the sequences only need minimal reprogramming to allow loading of user-provided pulses, greatly simplifying the workflow.

Originally the RF amplifiers were placed in the magnet room to reduce the cable length to the coil for three reasons, namely to reduce the cable losses, to use the impedance of the amplifiers for coil decoupling, and to enable a feedback loop for coil decoupling. With the feedback loop included, the amplifiers had an overall gain of about 90 dB. Together with the high in-room RF due to waves traveling out of the bore, this led to unwanted feedback, so the loop had to be disabled. Without the feedback loop, utilizing the amplifiers’ impedance for decoupling led to non-linear behavior depending on the reflected power, which in turn depends on the shim. Therefore, the circulators were included, also increasing the cable length by 7 m. Putting the amplifiers in the same space as the circulators was not possible because of space constraints and cooling constraints.

The overall losses between the amplifiers and the inputs of the elements of the body array amount to 2.79 dB. This includes 0.78 dB for the cables to and from the circulators, 0.15 dB for the circulators, 0.20 dB for the directional couplers, 0.58 dB for the cables on the bore liner (Aircell 5) and 1.1 dB for the T/R switches. This leads to approximately 520 W per element at the coil.

An improved version of the system that is being installed in Heidelberg now has the amplifiers outside the magnet room, with twice the amplifier peak power and cables with much lower attenuation to account for the increased cable length, leading to approximately 1.5 dB attenuation. This will result in roughly 900 W peak power per coil element. Moving the amplifiers outside the magnet room also facilitates cooling, since active cooling with fans directly at the amplifiers is possible and the duty cycle can therefore be increased.

The low transmit efficiency of integrated body arrays [32] is partly offset by the 32 kW total peak power provided by the amplifiers. With the shim shown in Fig 7, a *B*_*1*_^*+*^ of 13 μT was achieved in the center of the phantom. A dedicated shim for the center of the phantom using a local 8-channel transmit array comprised of the same transmit elements and 8 kW total peak RF power resulted in a maximum achievable *B*_*1*_^*+*^ of 12.4 μT [50], but covering a smaller field of view in z-direction [32]. A mean peak *B*_*1*_^*+*^ of 20 μT was reported in the prostate over 6 healthy volunteers for a close-fitting 16-channel loop-dipole array [51] as well as 13.2 μT for a 10-element array of fractionated dipoles [52]. Higher amplifier power for the integrated body array presented here would clearly be beneficial to improve transmission. Furthermore, a more efficient design for the body array could lead to further improvements, since the transmit elements in this project were not chosen for their efficiency, but for their good decoupling [40]; dipoles have been shown to be more efficient in integrated designs [53]. Using a more efficient coil design as proposed in [53] and increasing the amplifier peak output power to 2 kW per channel could increase the peak B1+ amplitude to 30 μT in the center of the body. Possible further improvements in addition to switching to dipoles as transmit elements include using T/R switches with reduced attenuation (or even excluding them and only using dedicated receive coils) and using cables with reduced attenuation on the bore liner.

Although the hardware of the system is conceived to apply low-power samples of the forward waveforms to the receive chain during transmit, this capability was not used in the current experiments. Instead, more conservative SAR limits based on amplitude-only supervision were utilized [37]. Future work will target real-time monitoring of both the amplitude and phase of the forward signal on all 32 channels, which represents a significant computational challenge but would further relax SAR restrictions.

Integrating a body array into the MRI system leaves more space in the bore, allowing for larger patients to be imaged, potentially with slim receive-only arrays, while local transmit arrays tend to take up quite some space in the bore. The large field of view allows imaging of large areas, which enables whole-body imaging with only a few stations. Among other applications, this new coil configuration might be beneficial for contrast agent free time-of-flight imaging of the lower extremities where local coils are known to limit the patient diameter [31]. First structural images show promising homogeneity and image contrast. In this study, the arms were placed outside the field of view because their high signal caused image artifacts due to their proximity to the coil elements. Since reception in this work was done with the body coil, a large SNR boost can be expected from local receive-only coils [54], also reducing the signal intensity differences between arms and torso. Ultimately, this would allow a workflow at UHF that mimics common practice at lower field strengths like 1.5 and 3 T.

Even though the DACs used in the presented systems are comparably slow and need up to 3 μs until the output is settled, the selective excitation examples show that 100 kHz sampling is possible. Imprecisions of the IQ modulators were successfully countered by using the system’s exciter in combination with the IQ modulators to achieve a coupled modulation strategy. Together with the large field of view, the capability for 32-channel selective excitation could potentially be used for reduced field of view imaging of, for example, large parts of the spinal column at UHF, which has already been shown for 16 transmit channels [55].

Since the add-on system does not affect the use of the system in standard configuration, all imaging protocols relying on the single-channel transmit system and the affiliated local Tx/Rx coils can still be used without any change, leading to an overall very versatile system. Future work will have to look more deeply into the capabilities, advantages and drawbacks of a 32-channel transmit system with an integrated body coil compared to standard systems with local transmit coils.

## Conclusion

We present a 32-channel transmit system add-on that is capable of 100 kHz sampling. Structural imaging with an integrated 32-channel body array is possible and selective excitation is shown. The system does not affect the operation of the original MRI system in single-channel mode. The presented add-on system provides a tool for large field of view imaging and a potentially more clinic-like workflow.
